# Complete Genome Sequences of Cluster F1 Mycobacteriophages Akhila and MilanaBonita

**DOI:** 10.1128/mra.01191-22

**Published:** 2022-12-20

**Authors:** Umer Ahsan, Nimra Ali, Chelsea Akintunde, Mohamed Ali, Caroline Bond, Brittney Canada, Soban Choudhry, Dennis Cortez, Nia Dennis, Tamia Gomez, Seerat Hameed, Daniel Higgins, Keisha Lacayo, Zeeshan Memon, Shubh Patel, Joel Philip, Ramon Polanco, Anshika Rajput, Malaika Raza, Anthony Recenello, Benjamin Scariah, Emmaline Seide, Stephany Shaji, Ariel Shalonov, Emaan Tariq, Zainab Tariq, Blessy Thomas, Rita Reddy, Fernando E. Nieto-Fernandez, Jillian C. Nissen

**Affiliations:** a Department of Biological Sciences, SUNY Old Westbury, Old Westbury, New York, USA; Portland State University

## Abstract

Akhila and MilanaBonita are mycobacteriophages that were isolated from soil in New York using Mycobacterium smegmatis. Both phages have genomes that are 56,251 bp long and contain 99 genes; the genomes differ by only 1 nucleotide. Based on gene content similarity to phages in the Actinobacteriophage Database, both phages are assigned to cluster F1.

## ANNOUNCEMENT

**FIG 1:**
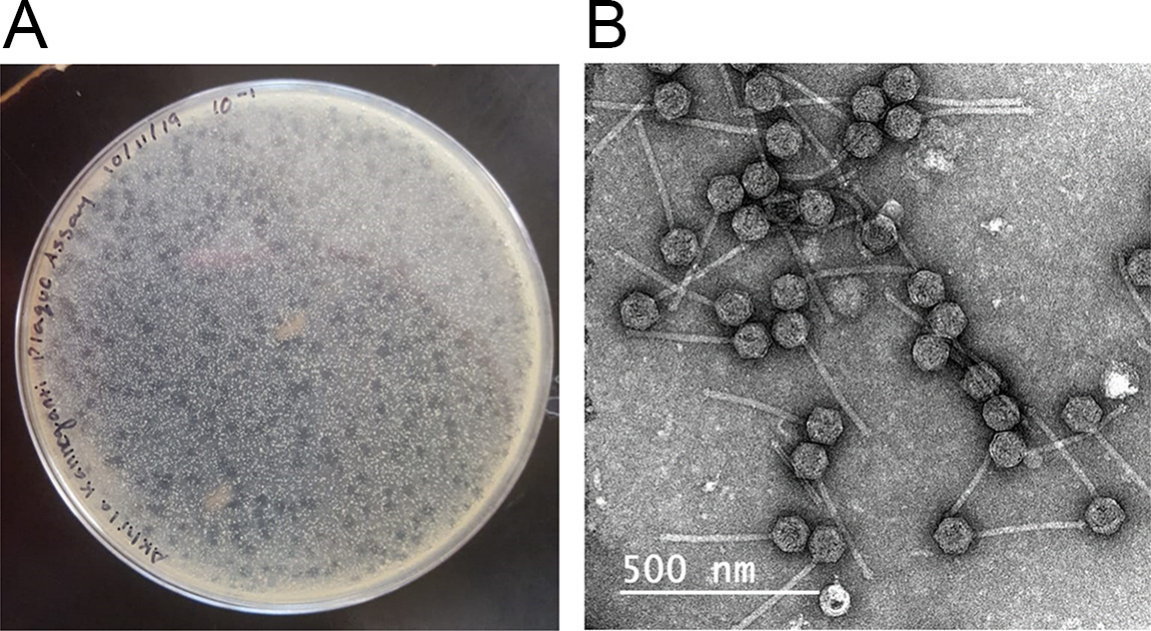
(A) Plaque morphology of Akhila. (B) Lysates of Akhila were mounted on Formvar-coated copper grids, stained with uranyl acetate (1%), and imaged with a JEOL JEM-2100 electron microscope at a magnification of ×20,000.

The phylum *Actinobacteria* consists of several human pathogens, notably members of the genus Mycobacterium (1). Bacteriophages are increasingly being considered as therapeutic agents to treat multidrug-resistant bacterial infections (2–4). Here, we describe the isolation and characterization of two mycobacteriophages, Akhila and MilanaBonita, which were isolated from soil samples collected on Long Island, New York (Table 1), in 2019 and 2018, respectively, using standard methods (5). In brief, soil samples were washed with 7H9 liquid medium, and bacteriophages were collected following filtration of the wash using a 0.22-μm-pore filter. This filtered wash was then inoculated with Mycobacterium smegmatis mc^2^155. Following incubation for 3 days at 37°C with shaking, the culture was centrifuged and filtered, and the filtrate was plated in a soft agar overlay containing M. smegmatis and incubated at 37°C. Both Akhila and MilanaBonita were purified through two rounds of plating and formed slightly turbid plaques (2.5 mm in diameter, with irregular edges) after 48 h at 37°C. Negative-staining transmission electron microscopy revealed that Akhila possesses a siphovirus morphology, with a capsid 86 to 89 nm in diameter and a tail 287 to 291 nm in length (*n* = 3) (Fig. 1).

**TABLE 1:** Soil sampling information, sequencing results, and genome characteristics for Akhila and MilanaBonita

Phage	Soil collection site coordinates	Soil collection date	Sequencing coverage (×)	Genome length (bp)	GC content (%)	Genome termini	No. of genes
Akhila	40.797324N, 73.572221W	September 2019	1,484	56,251	62.1	3′ single-stranded overhangs (5′-CGGAAGGCGC-3′)	99
MilanaBonita	40.86299N, 73.10535W	March 2018	176	56,251	62.1	3′ single-stranded overhangs (5′-CGGAAGGCGC-3′)	99

Phage DNA was isolated using the Promega Wizard DNA cleanup kit, prepared for sequencing using the NEBNext Ultra II library preparation kit, and sequenced on an Illumina MiSeq instrument with v3 reagents. Sequencing generated 585,578 and 70,313 single-end 150-bp reads for Akhila and MilanaBonita, respectively. Raw reads were assembled using Newbler v2.9, and Consed v29 was used to confirm that the genomes were complete and accurate and to determine phage termini (6). Sequencing results and genome characteristics are provided in Table 1. Notably, the genomes of the two phages differ by only 1 nucleotide, despite soil sample collection occurring at different locations (approximately 24.5 miles apart) in different years.

The genomes were annotated using DNA Master (http://cobamide2.bio.pitt.edu), PECAAN (https://discover.kbrinsgd.org), Glimmer v3.02 (7), GeneMark v2.5 (8), and Starterator v1.1. tRNA and transfer-messenger RNA (tmRNA) predictions were made using ARAGORN v1.2.38 (9) and tRNAscan-SE v3.0 (10). No tRNAs or tmRNAs were detected in either phage genome. Functional assignments were determined using BLASTp searches against the NCBI nonredundant database v2.13.0 and the Actinobacteriophage Database (11), HHpred (with the Conserved Domain Database [CDD], the SCOPe70 database, the Pfam-A database, and the Protein Data Bank [PDB]) (12), and Phamerator (13). All software was run using default parameters. Both phages contain 99 protein-coding genes, of which all but 9 are transcribed on one strand. Based on gene content similarity (GCS) of ≥35% to phages in the Actinobacteriophage Database (https://phagesdb.org), as determined using the Actinobacteriophage Database GCS tool, both phages are assigned to phage cluster F1. Consistent with phages in this cluster, Akhila and MilanaBonita are predicted to be temperate, encoding a predicted tyrosine integrase (gp37), a predicted immunity repressor (gp39), a predicted excise protein (gp42), and a predicted Cro protein (gp40). Both phages also contain a predicted mycobacteriophage mobile element 1 (MPME1) (gp74), which is present in some cluster F1 phages (14). The difference between the two phages is a single nucleotide substitution (which does not alter any gene start or stop site) within gene 19, which encodes a minor tail protein.

### Data availability.

Further information about phage characteristics is available at PhagesDB. The GenBank accession numbers are MW924649 for Akhila (https://phagesdb.org/phages/Akhila/) and MZ681519 for MilanaBonita (https://phagesdb.org/phages/MilanaBonita/), and the Sequence Read Archive (SRA) accession numbers are SRX14443480 for Akhila and SRX14597706 for MilanaBonita.
